# Effects of pressure support and pressure-controlled ventilation on lung damage in a model of mild extrapulmonary acute lung injury with intra-abdominal hypertension

**DOI:** 10.1371/journal.pone.0178207

**Published:** 2017-05-25

**Authors:** Cintia L. Santos, Raquel S. Santos, Lillian Moraes, Cynthia S. Samary, Nathane S. Felix, Johnatas D. Silva, Marcelo M. Morales, Robert Huhle, Marcelo G. Abreu, Alberto Schanaider, Pedro L. Silva, Paolo Pelosi, Patricia R. M. Rocco

**Affiliations:** 1 Laboratory of Pulmonary Investigation, Carlos Chagas Filho Institute of Biophysics, Federal University of Rio de Janeiro, Av. Carlos Chagas Filho, s/n, Bloco G-014, Ilha do Fundão, Rio de Janeiro, RJ, Brazil; 2 Laboratory of Experimental Surgery, Faculty of Medicine, Federal University of Rio de Janeiro, Av. Professor Rodolpho Paulo Rocco, 225, Ilha do Fundão, Rio de Janeiro, RJ, Brazil; 3 Laboratory of Cellular and Molecular Physiology, Carlos Chagas Filho Institute of Biophysics, Federal University of Rio de Janeiro, Av. Carlos Chagas Filho, s/n, Bloco G2-048, Ilha do Fundão, Rio de Janeiro, RJ, Brazil; 4 Department of Anesthesiology and Intensive Care Therapy, Pulmonary Engineering Group, University Hospital Carl Gustav Carus, Dresden University of Technology, Fetschertsrasse 74, Dresden, Germany; 5 IRCCS AOU San Martino-IST, Department of Surgical Sciences and Integrated Diagnostics, University of Genoa, Largo Rosanna Benzi 8, Genoa, Italy; Hospital for Sick Children, CANADA

## Abstract

Intra-abdominal hypertension (IAH) may co-occur with the acute respiratory distress syndrome (ARDS), with significant impact on morbidity and mortality. Lung-protective controlled mechanical ventilation with low tidal volume and positive end-expiratory pressure (PEEP) has been recommended in ARDS. However, mechanical ventilation with spontaneous breathing activity may be beneficial to lung function and reduce lung damage in mild ARDS. We hypothesized that preserving spontaneous breathing activity during pressure support ventilation (PSV) would improve respiratory function and minimize ventilator-induced lung injury (VILI) compared to pressure-controlled ventilation (PCV) in mild extrapulmonary acute lung injury (ALI) with IAH. Thirty Wistar rats (334±55g) received *Escherichia coli* lipopolysaccharide intraperitoneally (1000μg) to induce mild extrapulmonary ALI. After 24h, animals were anesthetized and randomized to receive PCV or PSV. They were then further randomized into subgroups without or with IAH (15 mmHg) and ventilated with PCV or PSV (PEEP = 5cmH_2_O, driving pressure adjusted to achieve tidal volume = 6mL/kg) for 1h. Six of the 30 rats were used for molecular biology analysis and were not mechanically ventilated. The main outcome was the effect of PCV *versus* PSV on mRNA expression of interleukin (IL)-6 in lung tissue. Regardless of whether IAH was present, PSV resulted in lower mean airway pressure (with no differences in peak airway or peak and mean transpulmonary pressures) and less mRNA expression of biomarkers associated with lung inflammation (IL-6) and fibrogenesis (type III procollagen) than PCV. In the presence of IAH, PSV improved oxygenation; decreased alveolar collapse, interstitial edema, and diffuse alveolar damage; and increased expression of surfactant protein B as compared to PCV. In this experimental model of mild extrapulmonary ALI associated with IAH, PSV compared to PCV improved lung function and morphology and reduced type 2 epithelial cell damage.

## Introduction

Intra-abdominal hypertension (IAH) is a clinical condition characterized by intra-abdominal pressure (IAP) ≥ 12 mmHg. Among other causes, it may result from sepsis, intra-abdominal bleeding, or trauma, and is associated with worse outcomes in these conditions [1,2,3,4]. IAH is highly prevalent in critically ill patients, affecting up to 64% of this population, and has a major impact on the function of the lungs and peripheral organs [3,5]. In the presence of preexisting alveolar-capillary damage, IAH promotes lung injury [6,7,8], edema, and increased intra-thoracic pressures, leading to atelectasis, airway closure, and deterioration of gas exchange [6]. Controlled mechanical ventilation with low tidal volume and optimization of positive end-expiratory pressure (PEEP), combined with neuromuscular blockade, has been recommended as a strategy to minimize ventilator-induced lung injury (VILI) [4,9]. However, IAH has been shown to potentiate dorsal atelectasis formation [6], and the relaxation of the respiratory muscles during controlled mechanical ventilation allows further cephalad displacement of the diaphragm, predominately in the ventral regions.

In experimental acute lung injury (ALI) [10,11], it has been demonstrated that pressure support ventilation (PSV) improves gas exchange and hemodynamics and prevents VILI as compared to controlled mechanical ventilation. On the other hand, PSV may lead to further lung injury if the inspiratory transpulmonary pressure and effort are excessively high [12,13,14]. So far, however, no study has compared the impact of PSV and pressure-controlled ventilation (PCV) on lung damage in experimental ALI with IAH.

Within this context, the present study was designed to test the hypothesis that, when delivered at a protective tidal volume, PSV compared to PCV would improve respiratory function, reduce the amount of collapsed areas in the lung, and prevent VILI in experimental extrapulmonary mild ALI with IAH. Part of the results of this study were published previously as an abstract [15].

## Material and methods

This study was carried out in strict accordance with the recommendations in the Guide for the Care and Use of Laboratory Animals of the National Institutes of Health. The protocol was approved by the Committee on the Ethics of Animal Experiments of the Health Science Center, Federal University of Rio de Janeiro, Rio de Janeiro, Brazil (CEUA: 019). All efforts were made to minimize suffering.

### Animal preparation and experimental protocol

Thirty Wistar rats (weight 334±55 g) received *Escherichia coli* O55:B5 lipopolysaccharide (LPS) (Sigma Chemical Co., St. Louis, MO, USA) intraperitoneally (i.p.) at a dose of 1,000 μg, suspended in saline solution to a total volume of 1,000 μL [16,17], to induce mild extrapulmonary ALI. After 24 h, animals were premedicated with 10 mg/kg diazepam i.p. (Compaz, Cristália, Itapira, SP, Brazil), followed by 100 mg/kg ketamine i.p. (Ketamin-S+, Cristália, Itapira, SP, Brazil) and 2 mg/kg midazolam i.p. (Dormicum, União Química, São Paulo, SP, Brazil). Following local anesthesia with 2% lidocaine (0.4 mL), a midline neck incision and tracheostomy were performed. Six of the 30 rats were used for molecular biology analysis and were not mechanically ventilated (non-ventilated, NV).

An intravenous (i.v.) catheter (Jelco 24G, Becton, Dickinson and Company, New Jersey, NJ, USA) was inserted into the tail vein, and anesthesia induced and maintained with midazolam (2 mg/kg/h) and ketamine (50 mg/kg/h). Additionally, 10 mL/kg/h Ringer’s lactate (B. Braun, Crissier, Switzerland) was administered i.v. in all groups. A second catheter (PE-50, Becton, Dickinson and Company) was then placed in the right internal carotid artery for blood sampling and gas analysis (Radiometer ABL80 FLEX, Copenhagen NV, Denmark), as well as monitoring of mean arterial pressure (MAP) (Networked Multiparameter Veterinary Monitor LifeWindow 6000 V; Digicare Animal Health, Boynton Beach, FL, USA). A 30-cm-long water-filled catheter (PE-205, Becton, Dickinson and Company) with side holes at the tip, connected to a differential pressure transducer (UT-PL-400, SCIREQ, Montreal, QC, Canada), was used to measure the esophageal pressure (Pes). The catheter was passed into the stomach and then slowly returned into the esophagus; its proper positioning was assessed with the “occlusion test” [18]. Heart rate (HR), MAP, intra-abdominal pressure (IAP), and rectal temperature were continuously monitored (Networked Multiparameter Veterinary Monitor LifeWindow 6000V, Digicare Animal Health, Florida, USA). Body temperature was maintained at 37.5±1°C using a heating bed. Colloids (Gelafundin^®^, B. Braun, São Gonçalo, RJ, Brazil) was administered intravenously (i.v., in 0.5-mL increments) as needed to maintain MAP> 60 mmHg.

Animals were mechanically ventilated (Servo-i, MAQUET, Solna, Sweden) in PCV or PSV (flow triggering) with PEEP = 5 cmH_2_O and FiO_2_ = 0.4. During PCV, animals were paralyzed with pancuronium bromide (2 mg/kg, i.v.). In both PCV and PSV, the driving pressure was adjusted to achieve V_T_ = 6 mL/kg. Following this step (5 min), animals were randomized to normal intra-abdominal pressure (nIAP) or intra-abdominal hypertension (IAH) subgroups. To induce IAH, a midline laparotomy 3 cm in length was performed to expose the abdominal cavity, and 15-cm hydrophilic gauze packs (Cremer, Blumenau, SC, Brazil) were placed in its four quadrants (one pack per quadrant). A catheter (PE-240) was inserted into the peritoneum for continuous IAP measurement [7,8,19], and a 2–0 silk suture was used to tie the catheter in place and ensure there was no leak. Both layers of the abdominal cavity were closed with 3–0 monofilament nylon suture (Ethilon®, São Paulo, SP, Brazil), which was tightened to maintain an IAP of 15 mmHg [7,8]. IAP was kept at this level throughout the experiment. In the nIAP group, a sham surgery was performed with the same technique used in the IAH group and manipulation of the abdominal cavity for the same amount of time, but no packing. Animals were kept on the same ventilator settings described above (PSV or PCV).

**Fig 1:**
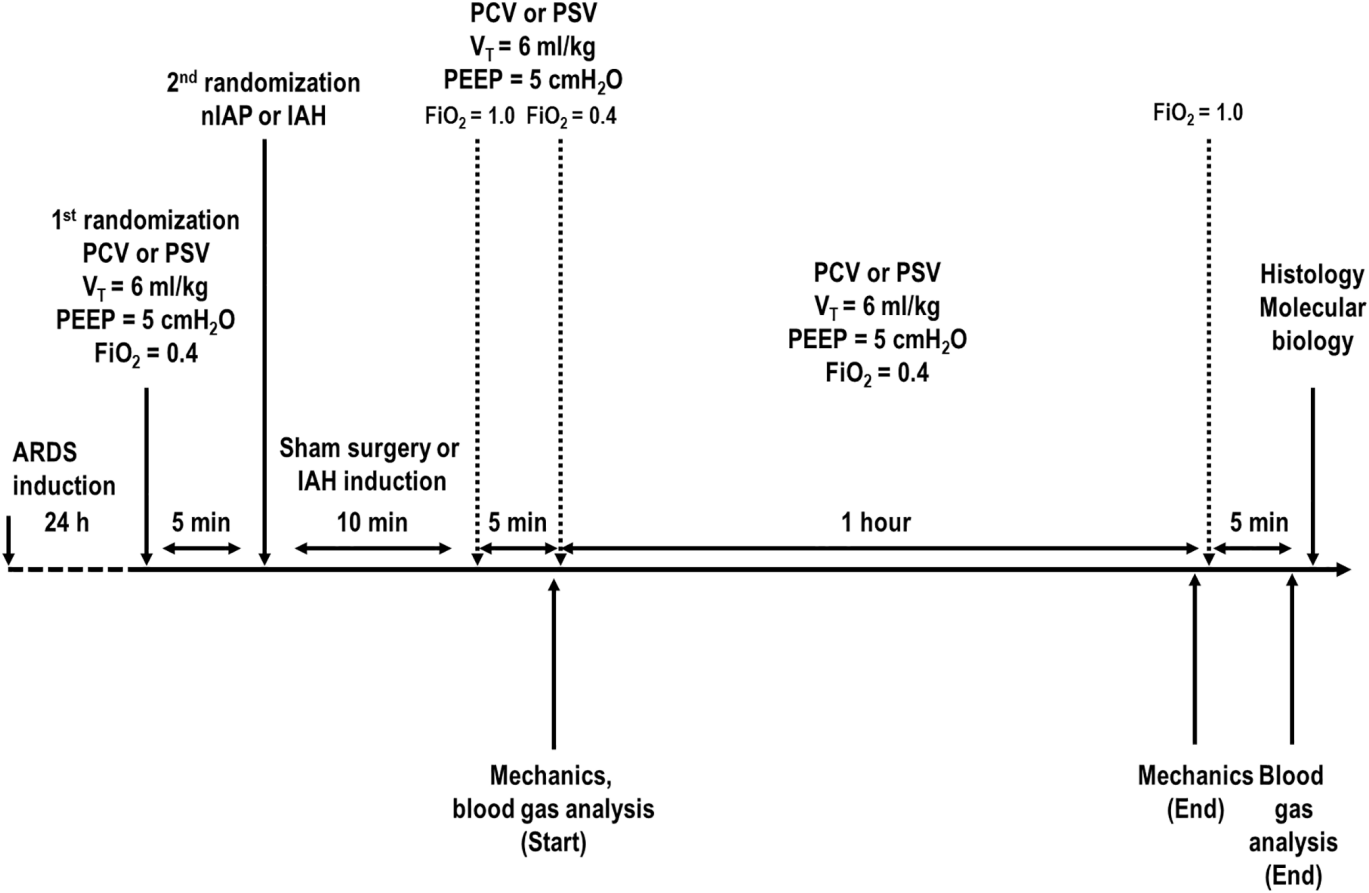
Timeline representation of the experimental protocol. First randomization: pressure-controlled ventilation (PCV) or pressure support ventilation (PSV). Second randomization: intra-abdominal hypertension (IAH) or normal intra-abdominal pressure (nIAP). Start: immediately after surgery (Sham) or IAH induction at PCV or PSV. V_T_, tidal volume; PEEP, positive-end expiratory pressure; FiO_2_, fraction of inspired oxygen. Mechanics and arterial blood gases were evaluated at Start and End (after 1 h of mechanical ventilation in PCV or PSV).

Arterial blood gases and respiratory system and lung mechanics were measured immediately after surgery and after 1 h of mechanical ventilation with PSV or PCV (End) (Fig 1). FiO_2_ was set at 1.0 and, after 5 min, arterial blood (300 μL) was drawn into a heparinized syringe to determine arterial oxygen partial pressure (PaO_2_), arterial carbon dioxide partial pressure (PaCO_2_), and arterial pH (pHa) (Radiometer ABL80 FLEX, Copenhagen NV, Denmark). Animals were then killed with sodium thiopental 60 mg/kg i.v., and their lungs extracted at PEEP = 5 cmH_2_O for lung histology and molecular biology analysis (Fig 1).

Airflow, airway pressure (P_aw_), and esophageal pressure (P_es_) were continuously recorded throughout the experiments on a computer running customer-made software written in LabVIEW (National Instruments, Austin, TX) [20,21]. V_T_ was calculated by digital integration of the airflow signal. Transpulmonary pressure (P,_L_) was calculated during inspiration and expiration as the difference between P_aw_ and P_es_. Peak and mean airway pressures (P_peak,aw_ and P_mean,aw_), transpulmonary pressures (P_peak,L_ and P_mean,L_), and the esophageal pressure generated 100 ms after onset of inspiratory effort (P_0.1_) were calculated. The respiratory rate (RR) was calculated from the P_es_ swings as the frequency per minute of each type of breathing cycle. The pressure–time product (PTP) per breath was calculated as the integral of ΔP_es_ over time [10,11,22,23]. The asynchrony index was calculated as the total number of asynchronous breaths divided by the total number of triggered and untriggered breaths, multiplied by 100 [22]. All signals were amplified in a four-channel signal conditioner (SC-24, SCIREQ, Montreal, QC, Canada), and sampled at 200 Hz with a 12-bit analog-to-digital converter (National Instruments; Austin, Texas, USA). All mechanical data were computed offline by a routine written in MATLAB (Version R2007a; The Mathworks Inc, Natick, Massachusetts, USA).

### Histology

#### Light microscopy

A laparotomy was performed immediately after blood sampling at the end of experiments. Heparin (1,000 IU) was injected into the tail vein. The trachea was then clamped at end-expiration (PEEP = 5 cmH_2_O) and the abdominal aorta and vena cava were sectioned, yielding a massive hemorrhage that quickly killed the animals. The lungs were removed *en bloc*. The left lung was frozen in liquid nitrogen, immersed in Carnoy’s solution, embedded in paraffin, cut longitudinally at the level of the central zone into slices of 4 μm thickness, and stained with hematoxylin-eosin for histological analysis [22]. Photomicrographs at magnifications of ×100, ×200 and ×400 were obtained from eight non-overlapping fields of view per section under a light microscope (Olympus BX51, Olympus Latin America-Inc., Brazil). Diffuse alveolar damage (DAD) was quantified using a weighted scoring system by a researcher blinded to the experimental protocol [24]. Briefly, scores of 0 to 4 were used to represent alveolar collapse, interstitial edema, and septal thickening with 0 standing for no effect and 4 for maximum severity. Additionally, the extent of each scored characteristic per field of view was determined on a scale of 0 to 4, with 0 standing for no visible evidence and 4 for complete involvement. Scores were calculated as the product of severity and extent of each feature, and thus ranged from 0 to 16. The cumulative DAD score was calculated as the sum of each score characteristic, and ranged from 0 to 48. Scoring was assessed independently by two co-authors (J.D.S. and C.S.S.) who are experts in lung pathology. Both assessors were blinded to group assignment. The scores of each expert were combined to yield a final score by arithmetic averaging.

### Biological markers of inflammation, fibrogenesis, alveolar stretch, and epithelial and endothelial cell damage

Quantitative real-time reverse transcription polymerase chain reaction (RT-PCR) was performed to measure biomarkers associated with inflammation (interleukin [IL]-6), fibrogenesis (type III procollagen), alveolar stretch (amphiregulin), type II alveolar cell mechanotransduction (surfactant protein [SP]-B), and endothelial cell injury (vascular cellular adhesion molecule [VCAM]-1). The primers used are described in the online supplement (Additional file 1: S1 Table). Central slices of the right lung were cut, collected in cryotubes, flash-frozen by immersion in liquid nitrogen, and stored at −80°C. Total RNA was extracted from frozen tissues using the RNeasy Plus Mini Kit (Qiagen, Hilden, Germany), in accordance with the manufacturer’s recommendations. RNA concentrations were measured by spectrophotometry in a Nanodrop ND-1000 system (ThermoScientific, Wilmington, DE, USA). First-strand cDNA was synthesized from total RNA using a Quantitec reverse transcription kit (Qiagen, Hilden, Germany). Relative mRNA levels were measured with a SYBR green detection system in an ABI 7500 real-time PCR system (Applied Biosystems, Foster City, California, USA). Samples were run in triplicate. For each sample, the expression of each gene was normalized to the acidic ribosomal phosphoprotein P0 (*36B4*) housekeeping gene [25] and expressed as fold change relative to respective NV animals, using the 2^–ΔΔCt^ method, where ΔCt = Ct (reference gene)–Ct (target gene) [26].

### Statistical analysis

Sample size calculation was based on pilot studies and on previous studies in rodents using similar ventilator settings [22,27]. A sample size of six animals per group would provide the appropriate power (1 − β = 0.8) to identify significant (α = 0.05) differences in the percentage of IL-6 between PCV and PSV, during nIAP, taking into account an effect size d = 1.38, a two-sided test, and a sample size ratio = 1 (G*Power 3.1.9.2, University of Düsseldorf, Düsseldorf, Germany).

Data were tested for normality using the Kolmogorov-Smirnov test with Lilliefors’ correction, while the Levene median test was used to evaluate the homogeneity of variances. If both conditions were satisfied, the effects of different ventilatory strategies (PCV and PSV) in nIAP and IAH were analyzed by using two-way repeated measures ANOVA followed by Bonferroni’s test. One-way ANOVA on ranks followed by Dunn’s post-hoc test was employed to compare lung histology and molecular biology data. Parametric data were expressed as mean ± SD, while non-parametric data were expressed as median (interquartile range). The significance level was set at p = 0.05. All tests were performed in GraphPad Prism version 6.01 (GraphPad Software, San Diego, CA).

## Results

Thirty animals were used, with 6 animals allocated to each group, including the NV group. No animals died during the experiments.

MAP was maintained above 60 mmHg throughout the experiments (Additional file 2: S2 Table). At Start, MAP was higher in PSV than PCV, regardless of nIAP or IAH. At End, the amount of fluid administered was higher in PCV than PSV (p<0.05), both in nIAP and IAH animals (Additional file 2: S2 Table).

Oxygenation improved significantly from Start to End in IAH animals ventilated with PSV. There were no significant differences in PaCO_2_ between PCV and PSV at Start and End, while pHa was lower in PCV than PSV animals (Table 1).

**Table 1:** Arterial blood gases at Start and End.

		nIAP		IAH	
	Time point	PCV	PSV	PCV	PSV
PaO_2_/FiO_2_	Start	304 ± 174	341 ± 155	280 ± 143	245 ± 146
	End	334 ± 115	429 ± 132	329 ± 112	421 ± 92*
pHa	Start	7.31 ± 0.08	7.31 ± 0.07	7.31 ± 0.04	7.35 ± 0.09
	End	7.22 ± 0.05*	7.31 ± 0.05#	7.20 ± 0.06*	7.30 ± 0.03#
PaCO_2_ (mmHg)	Start	44.9 ± 15.8	35.2 ± 14.0	46.6 ± 15.7	39.1 ± 12.4
	End	43.5 ± 17.5	39.8 ± 11.3	50.5 ± 16.8	47.7 ± 8.5

IAH, intra-abdominal hypertension; nIAP, normal intra-abdominal pressure; PCV, pressure-controlled ventilation; PSV, pressure support ventilation; Start, after sham surgery (nIAP) or IAH induction; End, nIAP or IAH after 1 h mechanical ventilation with PCV or PSV; PaO_2_/FiO_2_, arterial oxygen partial pressure divided by fraction of inspired oxygen; pHa, arterial pH; PaCO_2_, arterial carbon dioxide partial pressure. Comparisons were performed by two-way repeated-measures ANOVA followed by Bonferroni’s post-hoc test. Values are given as mean ± standard deviation of 6 animals/group.

*Significantly different from Start (p<0.05).

#Significantly different from PCV group at the corresponding time point (p<0.05).

At Start, mean V_T_ and RR were similar in all groups. PSV yielded lower P_mean,aw_ in nIAP and IAH animals compared to PCV. No significant differences were observed in P_peak,aw_, P_peak,L_, P_mean,L_, P_0.1_, PTP, or asynchrony index between PSV and PCV animals in the nIAP or IAH groups (Table 2).

**Table 2:** Respiratory parameters at Start and End.

Parameters	Time	nIAP		IAH	
		PCV	PSV	PCV	PSV
V_T_ (mL/kg)	Start	6.3 ± 0.6	5.9 ± 0.5	6.0 ± 0.5	5.8 ± 0.4
	End	5.9 ± 0.4	6.7 ± 1	5.6 ± 0.5	6.2 ± 0.5
RR (breaths/min)	Start	80 ± 0	62 ± 20	79 ± 2	77 ± 20
	End	80 ± 0	70 ± 20	80 ± 0	67 ± 30
P_peak,aw_ (cmH_2_O)	Start	13 ± 1	14 ± 4	16 ± 2	12 ± 4
	End	13 ± 1	13 ± 4	16 ± 2	12 ± 2
P_mean,aw_ (cmH_2_O)	Start	8.3 ± 0.7	6.8 ± 0.5#	9.1 ± 1.0	6.4 ± 0.7#
	End	8.0 ± 0.7	6.8 ± 0.9#	9.2 ± 0.7	6.2 ± 1.0#
P_peak,L_ (cmH_2_O)	Start	13 ± 1	16 ± 4	13 ± 3	14 ± 3
	End	12 ± 0.9	16 ± 5	13 ± 1	14 ± 2
P_mean,L_ (cmH_2_O)	Start	7.9 ± 0.7	7.5 ± 0.6	7.4 ± 1	7.4 ± 0.3
	End	7.4 ± 0.6	7.5 ± 2.0	7.4 ± 0.8	7.4 ± 1
P_0.1_ (cmH_2_O)	Start	-	0.28 ± 0.6	-	0.81 ± 0.6
	End	-	0.66 ± 0.4	-	0.94 ± 0.9
PTP (cmH_2_O*s)	Start	-	2.2 ± 0.7	-	1.4 ± 0.7
	End	-	2.1 ± 0.9	-	1.5 ± 0.8
Asynchrony index (%)	Start	-	3.9 ± 2.0	-	3.5 ± 2.0
	End	-	2.8 ± 2.0	-	1.3 ± 1.0

nIAP, normal intra-abdominal pressure, IAH, intra-abdominal hypertension; PCV, pressure-controlled ventilation; PSV, pressure support ventilation; Start, after sham surgery (nIAP) or IAH induction; End, nIAP or IAH after 1 h mechanical ventilation with PCV or PSV; V_T_, tidal volume; RR, respiratory rate; Ppeak,aw, peak airway pressure; Pmean,aw, mean airway pressure; Ppeak,L, peak transpulmonary pressure; Pmean,L, mean transpulmonary pressure; P_0.1_, driving pressure; PTP, pressure-time product. Comparisons were performed by two-way repeated-measures ANOVA followed by Bonferroni’s post-hoc test (p < 0.05). Values are given as mean ± standard deviation of 6 animals/group. #Significantly different from PCV group at the corresponding time point (p<0.05).

**Fig 2:**
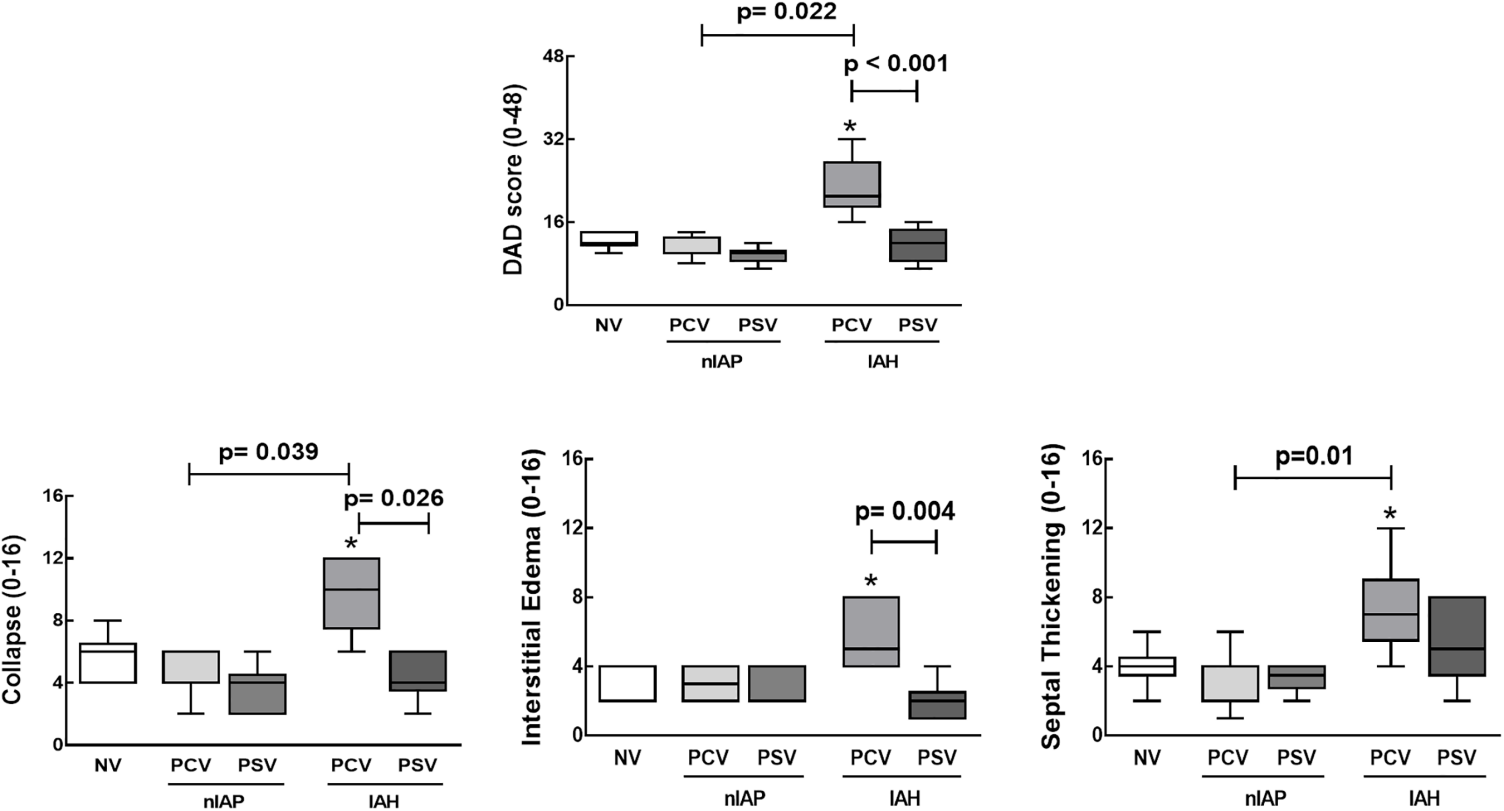
Cumulative DAD score (scores arithmetically averaged from two independent investigators) representing injury from alveolar collapse, interstitial edema, and septal thickening in animals with normal intra-abdominal pressure (nIAP) or intra-abdominal hypertension (IAH) mechanically ventilated in pressure-controlled ventilation (PCV) or pressure support ventilation (PSV) mode. NV: non-ventilated animals. Values are given as medians, interquartile ranges, and minimum/maximum of 6 animals in each group. Statistical significance was accepted at p < 0.05. *Significantly different from NV.

In nIAP animals, no significant differences were observed between PCV and PSV with regard to alveolar collapse, interstitial edema, septal thickening, or DAD score (Additional file 3: S1 Fig, Fig 2). However, in the IAH group, PSV was associated with less alveolar collapse, interstitial edema, and DAD compared to PCV (Fig 2). In the presence of IAH, alveolar collapse, interstitial edema, septal thickening, and DAD score were higher in PCV than NV.

**Fig 3:**
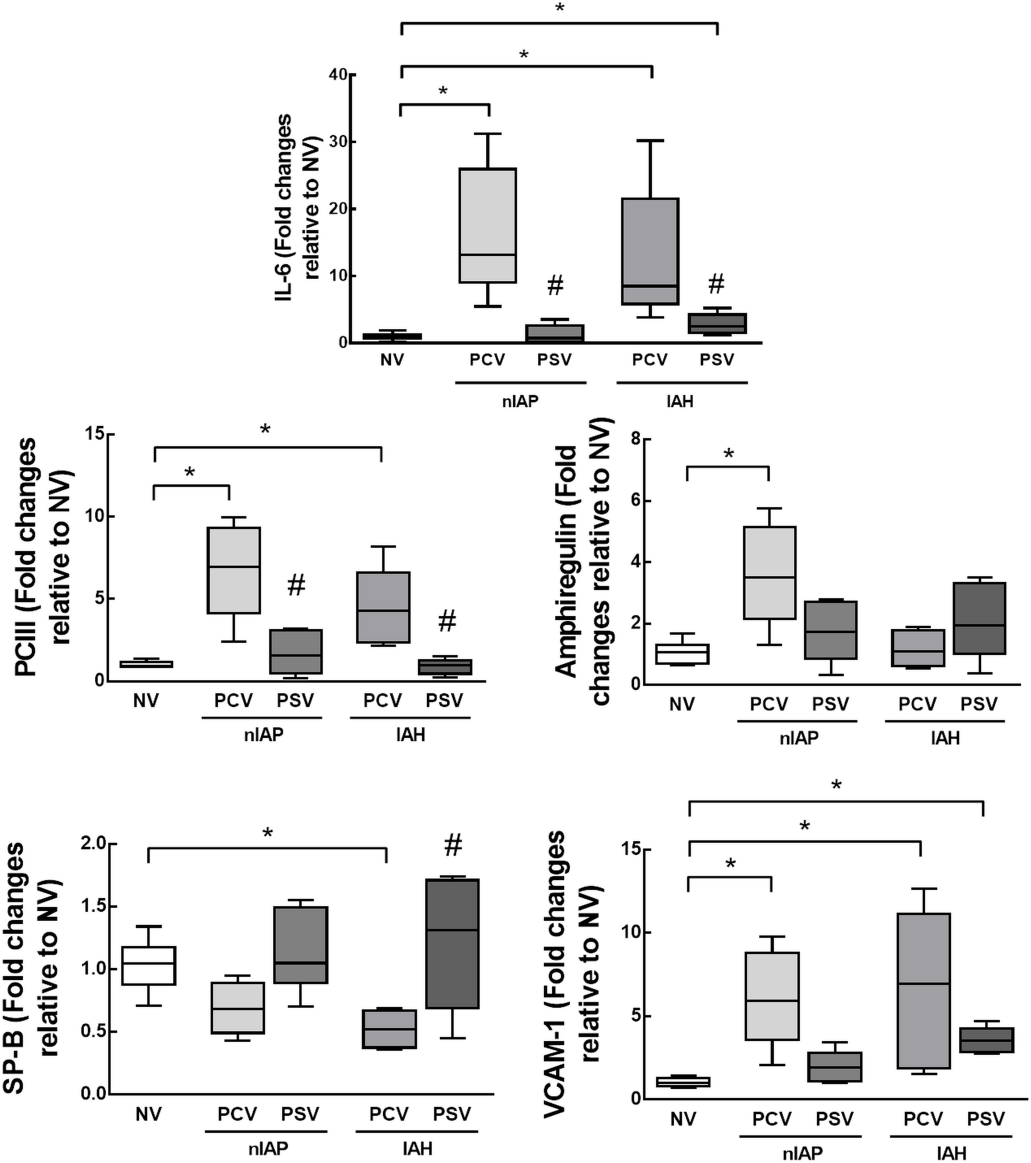
Real-time polymerase chain reaction analysis of biological markers associated with inflammation (interleukin [IL]-6), fibrogenesis (type III procollagen [PCIII]), pulmonary stretch (amphiregulin), type II epithelial cell damage (surfactant protein [SP]-B), and endothelial cell damage (vascular cellular adhesion molecule [VCAM-1]) in animals with normal intra-abdominal pressure (nIAP) or intra-abdominal hypertension (IAH) mechanically ventilated in pressure-controlled ventilation (PCV) or pressure support ventilation (PSV) mode. Values are given as medians, interquartile ranges, and minimum/maximum of 6 animals in each group. Relative gene expression was calculated as a ratio of average gene expression compared with the reference gene (*36B4*) and expressed as fold change relative to non-ventilated (NV) animals. *Significantly different from NV (p<0.05). #Significantly different from PCV (p<0.05).

Gene expression of biological markers associated with inflammation (IL-6), fibrogenesis (PCIII), pulmonary stretch (amphiregulin), and type II epithelial cell and endothelial cell damage (SP-B and VCAM-1 respectively) is illustrated in Fig 3. In both nIAP and IAH, IL-6, PCIII, and VCAM-1 expressions were higher in groups ventilated with PCV compared to NV. In nIAP, PCV was associated with higher amphiregulin expression than NV. In IAH, SP-B expression was lower in PCV than NV and higher in PSV than PCV, whereas VCAM-1 expression was higher in PSV than NV.

## Discussion

In the rat model of mild extrapulmonary ALI used in this study, we found that, regardless of IAP, PSV resulted in lower P_mean,RS_ (with no differences in P_peak,RS_, P_peak,L_, or P_mean,L_) and decreased mRNA expression of biomarkers associated with inflammation and fibrogenesis as compared with PCV. Specifically, in the presence of IAH, PSV improved oxygenation, increased expression of surfactant protein (SP)-B, and was associated with less alveolar collapse, interstitial edema, and alveolar damage than PCV.

To the best of our knowledge, this is the first study investigating the impact of PSV versus PCV on VILI in experimental ALI with IAH. The model of extrapulmonary ALI induced by *E*. *coli* LPS was chosen for this study because, in the clinical setting, IAH is most often associated with abdominal sepsis. Accordingly, 24 h after LPS administration, changes in lung histology, alterations in the alveolar-capillary barrier, inflammation, and physiologic dysfunction were observed, reproducing several of the main features of human ARDS [28]. In addition, at this time point, IAH was induced to simulate the course of increased IAP in critically ill patients with a model of extrapulmonary ALI [17,29]. IAH was induced by inserting gauze packs into the abdominal cavity until an IAP of 15 mmHg was achieved [7,8,19]. According to WSACS recommendations [30], IAH is defined as an IAP higher than 12 mmHg, and the average IAP in patients undergoing mechanical ventilation is 15 mmHg. Other methods used to mimic IAH, such as CO_2_/air inflation [31,32,33] and intraperitoneal fluid infusion [34], may interfere with the pathophysiological response to IAH or represent an additional physiologic variable altering the body’s response to IAH. The advantage of our IAH model was the maintenance of a high IAP without any side effects related to gas inflation of the peritoneal cavity.

In both PCV and PSV mode, animals were ventilated with a protective V_T_ (6 mL/kg) and PEEP = 5 cmH_2_O. In the presence of IAH, increased PEEP may have a deleterious impact on hemodynamics and may increase fluid and/or vasoactive drug requirements, with no effects on lung injury [8]. We chose to analyze mRNA expression of biomarkers associated with inflammation (IL-6), type II epithelial cell damage (SP-B), and endothelial cell damage (VCAM-1) in the lung because of the role of these substances as mediators in the pathogenesis of VILI [35,36]. The expression of PCIII mRNA in lung tissue was also evaluated because it this the first form of collagen to be remodeled in the course of lung fibrogenesis, and is an early marker of lung parenchymal remodeling [37]. Amphiregulin, in turn, was measured because its expression is positively modulated by hyperinflation, it activates chemokines, cytokines, and adhesion molecules, and represents a novel candidate gene in VILI [20,38].

IAH is a primary cause of organ dysfunction and abdominal compartment syndrome (ACS), a clinical entity which carries a high mortality rate and requires urgent, targeted intervention [39,40]. Over the years, several strategies have been developed to attempt to mitigate IAH and prevent its progression to ACS [41]. IAH does not only affect abdominal organs, but also has a great impact on respiratory function [2], with major practical consequences for mechanically ventilated patients [42]; high IAP makes it particularly difficult to satisfy the mandates of lung-protective mechanical ventilation while providing adequate oxygenation. The optimal mechanical ventilation settings for ARDS with IAH have yet to be determined. A protective ventilation strategy with a low V_T_ (6 mL/kg ideal body weight) and an airway plateau pressure of < 30 cm H_2_O has been demonstrated to improve survival in patients with ARDS [9]. Optimization of mechanical ventilation and recruitment [4], combined with neuromuscular blockade, has been proposed as a strategy to reduce IAP in patients with IAH or ACS [43]. Research suggests the optimal ventilator management of patients with ARDS and IAH should include the following: (a) monitoring of IAP, P_es_, and hemodynamic parameters; (b) ventilation with protective V_T_, recruitment maneuvers, and PEEP set according to the “best” compliance of the respiratory system or lung; (c) deep sedation (with or without neuromuscular blockade in severe ARDS); and (d) an open abdomen in selected patients with severe ACS [44]. Previous experimental studies investigating the optimization of mechanical ventilation during IAH were performed with controlled mechanical ventilation and mainly focused on respiratory mechanics, partitioned into its lung and chest wall components, and/or gas exchange in healthy and diseased animals [31,32]; others yet focused on hemodynamics [45,46].

Assisted mechanical ventilation has been proposed as a potential alternative to controlled mechanical ventilation, with advantages of better alveolar recruitment and gas exchange with less hemodynamic impairment, muscle atrophy, and lung injury in experimental ARDS [10,11,47,48,49]. However, in severe ARDS, high spontaneous inspiratory effort may also lead to increased lung injury due to abnormally high inspiratory activity and transpulmonary pressure [12,13].

Our data suggest that, in the presence of mild extrapulmonary ARDS with IAH, PSV with moderate PEEP reduced atelectasis, likely due to moderate activation of respiratory muscles (and, in particular, the diaphragm) [50,51,52]. This reduction in atelectasis was associated with oxygenation improvement from Start to End in the PSV group, probably due to increased blood flow distribution [53]. However, the increase in transpulmonary pressure associated with PSV may also result in further lung damage [14,54,55], as lungs with pre-existing damage are more susceptible to increased stress, and regional changes in transpulmonary pressure may be associated with *pendelluft* (movement of air from more recruited regions to less recruited regions during early inspiration without a gain in tidal volume) [56]. Additionally, negative pleural pressures have been shown to yield negative alveolar pressures and increased vascular pressure, thus worsening lung edema [55].

In the presence of IAH, we cannot rule out that the beneficial effects of PSV on lung histology, decreasing DAD, atelectasis, and interstitial edema were associated with the reduced amount of fluids administered during mechanical ventilation [57]. The reduction in atelectasis was not associated with a decrease in P_peak,L_, which suggests that transpulmonary pressure might not be the only parameter influencing alveolar recruitment. Our data suggest that, in the presence of IAH, transpulmonary pressure is more effective to reduce atelectasis when induced by decreased pleural pressure than by increased airway pressure [58]. Even though DAD score was similar in the PCV and PSV groups in nIAP, biological markers differed according to mechanical ventilation strategy. In both the IAH and the nIAP groups, PSV was also associated with decreased mRNA expression of biological lung tissue markers associated with inflammation and fibrogenesis compared to PCV, while in IAH specifically, SP-B expression was higher with PSV than with PCV, thus suggesting less type II epithelial cell damage. The main mechanisms leading to lung injury may be associated to [10]: 1) peak airway and transpulmonary pressure (i.e., stress); 2) respiratory rate and minute ventilation; 3) shear stress due to continuous opening and closing of collapsed alveoli during tidal breathing; 4) mean airway and transpulmonary pressures (i.e., static strain); 5) regional stress and strain (transpulmonary pressures); and 6) redistribution of perfusion from collapsed towards aerated lung regions. In the present study, total inspiratory stress, respiratory rate, and minute ventilation were comparable between PSV and PCV. However, different distributions of forces leading to the same transpulmonary stress pressure can play a role. PSV only reduced atelectasis in the IAH group; thus, shear stress may not fully explain the reduction in lung injury observed during assisted ventilation. Mean airway pressure was lower during PSV both with and without IAH. The present study thus suggests that reductions in static stress and strain may markedly affect lung injury in the context of PSV [59]. Finally, we cannot exclude that redistribution in perfusion from collapsed towards aerated lung regions, which likely occurred at least in the IAH group, might have contributed partly to reductions in lung injury.

Although VCAM-1 mRNA expression was increased in animals ventilated with PCV (in both nIAP and IAH) and PSV (in IAH) compared to NV, interstitial edema was more pronounced in PCV than in either NV or PSV in IAH. This apparent dissociation between morphological and molecular data may be attributed to the fact that VCAM-1 was evaluated at the RNA level instead of the protein level, and it takes time to observe the consequences of endothelial dysfunction in lung morphology. Moreover, interstitial edema is associated not only with endothelial cell dysfunction but also with lung perfusion distribution, which may differ according to mechanical ventilation strategy [60] and to inspiratory effort during PSV [54].

PSV was associated with asynchrony indices of 3–4%, suggesting that, within a minimal threshold limit as recommended (10%) [23,61], asynchronies do not seem to play a relevant role in promotion of lung injury. In line with our results, previous experimental studies using mild ARDS models without IAH reported a reduction in lung injury when assisted ventilation was compared to PCV [10,11,49]. In contrast, a previous study in healthy pigs with IAH at 30 mmHg reported greater histopathological damage to the lungs with assisted ventilation than with controlled mechanical ventilation [62].

### Limitations

This study has several limitations. First, we used a specific model of mild extrapulmonary ALI induced by intraperitoneal endotoxin injection. Thus, our results may not be extrapolated to other ALI models in small or large animals, nor to severe ALI. Second, we did not assess possible long-term effects of PSV, nor did we assess other types of assisted ventilation in the setting of IAH. Technical limitations include the fact that a specific level of IAH (15 mmHg) was used, and that mediators were measured in lung tissue, but not in blood. Third, higher IAP may lead to increased pulmonary artery pressure (PAP) [63,64]. Pulmonary arterial pressure was not measured due to technical difficulties faced when inserting catheter into pulmonary artery while keeping the animal alive without hindering our primary hypothesis (PSV versus PCV in ALI with IAH). Future studies are required to investigate the cardiopulmonary interaction during IAH using different ventilation strategies. Fourth, even though it is important to exclude procedure-related issues, we were unable to keep ALI animals with IAH alive during 1 hour spontaneous breathing. Lastly, since the observation time was relatively short (1 h mechanical ventilation), the expression of mediators was quantified using RT-PCR instead of ELISA. It is well known that 1 h is sufficient time to produce changes in mRNA expression, but not to significantly change levels of protein [7,8,10,11,20]. Keeping small ALI animals alive in the presence of increased IAH and mechanical ventilation (PCV or PSV) during longer periods of time would have required higher amounts of fluids, occasional use of vasoactive drugs to maintain MAP > 60 mmHg, and bicarbonate for metabolic acidosis. All these therapeutic strategies might have interfered with individual gene activation, thus hindering assessment of our primary hypothesis.

## Conclusion

In the model of mild ALI with IAH used in this study, PSV was associated with less atelectasis, interstitial edema, diffuse alveolar damage, and biological markers of inflammation, fibrogenesis, and type II epithelial cell damage than PCV.

## Supporting information

S1 Table Forward and reverse oligonucleotide sequences of target gene primers. Primers used in experiments. IL-6, interleukin-6; PCIII, pro-collagen III; SP-B, surfactant protein B; VCAM-1, vascular cell adhesion molecule-1; *36B4*, acidic ribosomal phosphoprotein P0.
(DOCX)

S2 Table Amount of fluids administered and mean arterial pressure. (DOCX)

S1 Fig Photomicrographs of lung parenchyma stained with hematoxylin-eosin. Original magnification ×200. Arrows: alveolar collapse. AD: alveolar duct. nIAP: animals with normal intra-abdominal pressure. IAH: animals with intra-abdominal hypertension. PCV: mechanically ventilated in pressure-controlled ventilation. PSV: mechanically ventilated with pressure support ventilation. NV: non-ventilated animals.
(DOCX)

## References

[1] Malbrain ML, Chiumello D, Pelosi P, Bihari D, Innes R, Ranieri VM, et al (2005) Incidence and prognosis of intraabdominal hypertension in a mixed population of critically ill patients: a multiple-center epidemiological study. Crit Care Med 33: 315–322. 15699833 10.1097/01.ccm.0000153408.09806.1b

[2] Malbrain ML, Chiumello D, Cesana BM, Reintam Blaser A, Starkopf J, Sugrue M, et al (2014) A systematic review and individual patient data meta-analysis on intra-abdominal hypertension in critically ill patients: the wake-up project. World initiative on Abdominal Hypertension Epidemiology, a Unifying Project (WAKE-Up!). Minerva Anestesiol 80: 293–306. 24603146

[3] Vidal MG, Ruiz Weisser J, Gonzalez F, Toro MA, Loudet C, Balasini C, et al (2008) Incidence and clinical effects of intra-abdominal hypertension in critically ill patients. Crit Care Med 36: 1823–1831. doi: 10.1097/CCM.0b013e31817c7a4d 18520642 10.1097/CCM.0b013e31817c7a4d

[4] Kirkpatrick AW, Roberts DJ, De Waele J, Jaeschke R, Malbrain ML, De Keulenaer B, et al (2013) Intra-abdominal hypertension and the abdominal compartment syndrome: updated consensus definitions and clinical practice guidelines from the World Society of the Abdominal Compartment Syndrome. Intensive Care Med 39: 1190–1206. doi: 10.1007/s00134-013-2906-z 23673399 10.1007/s00134-013-2906-z PMC3680657

[5] Vivier E, Metton O, Piriou V, Lhuillier F, Cottet-Emard JM, Branche P, et al (2006) Effects of increased intra-abdominal pressure on central circulation. Br J Anaesth 96: 701–707. doi: 10.1093/bja/ael071 16595615 10.1093/bja/ael071

[6] Quintel M, Pelosi P, Caironi P, Meinhardt JP, Luecke T, Herrmann P, et al (2004) An increase of abdominal pressure increases pulmonary edema in oleic acid-induced lung injury. Am J Respir Crit Care Med 169: 534–541. doi: 10.1164/rccm.200209-1060OC 14670801 10.1164/rccm.200209-1060OC

[7] Santos CL, Moraes L, Santos RS, Oliveira MG, Silva JD, Maron-Gutierrez T, et al (2012) Effects of different tidal volumes in pulmonary and extrapulmonary lung injury with or without intraabdominal hypertension. Intensive Care Med 38: 499–508. doi: 10.1007/s00134-011-2451-6 22234736 10.1007/s00134-011-2451-6

[8] Santos CL, Moraes L, Santos RS, dos Santos Samary C, Silva JD, Morales MM, et al (2014) The biological effects of higher and lower positive end-expiratory pressure in pulmonary and extrapulmonary acute lung injury with intra-abdominal hypertension. Crit Care 18: R121 doi: 10.1186/cc13920 24928415 10.1186/cc13920 PMC4095606

[9] Putensen C, Theuerkauf N, Zinserling J, Wrigge H, Pelosi P (2009) Meta-analysis: ventilation strategies and outcomes of the acute respiratory distress syndrome and acute lung injury. Ann Intern Med 151: 566–576. 19841457 10.7326/0003-4819-151-8-200910200-00011

[10] Saddy F, Moraes L, Santos CL, Oliveira GP, Cruz FF, Morales MM, et al (2013) Biphasic positive airway pressure minimizes biological impact on lung tissue in mild acute lung injury independent of etiology. Crit Care 17: R228 doi: 10.1186/cc13051 24103805 10.1186/cc13051 PMC4057608

[11] Saddy F, Oliveira GP, Garcia CS, Nardelli LM, Rzezinski AF, Ornellas DS, et al (2010) Assisted ventilation modes reduce the expression of lung inflammatory and fibrogenic mediators in a model of mild acute lung injury. Intensive Care Med 36: 1417–1426. doi: 10.1007/s00134-010-1808-6 20333356 10.1007/s00134-010-1808-6

[12] Yoshida T, Roldan R, Beraldo MA, Torsani V, Gomes S, De Santis RR, et al (2016) Spontaneous Effort During Mechanical Ventilation: Maximal Injury With Less Positive End-Expiratory Pressure. Crit Care Med 44: e678–688. doi: 10.1097/CCM.0000000000001649 27002273 10.1097/CCM.0000000000001649

[13] Yoshida T, Fujino Y, Amato MB, Kavanagh BP (2016) Spontaneous Breathing During Mechanical Ventilation—Risks, Mechanisms & Management. Am J Respir Crit Care Med. 10.1164/rccm.201604-0748CP 27786562

[14] Brochard L, Slutsky A, Pesenti A (2017) Mechanical Ventilation to Minimize Progression of Lung Injury in Acute Respiratory Failure. American journal of respiratory and critical care medicine 195: 438–442. doi: 10.1164/rccm.201605-1081CP 27626833 10.1164/rccm.201605-1081CP

[15] Santos CL SR, Moraes L, Samary CS, Felix NS, Fiorio Júnior PL, Morales MM, Abreu MG, Pelosi P, Schanaider A, Silva PL, Rocco PR (2015) Effects of pressure control and pressure support ventilation on ventilator induced lung injury in experimental acute respiratory distress syndrome with intra-abdominal hypertension. Intensive Care Medicine Experimental 3: A806.

[16] Riva DR, Oliveira MB, Rzezinski AF, Rangel G, Capelozzi VL, Zin WA, et al (2008) Recruitment maneuver in pulmonary and extrapulmonary experimental acute lung injury. Critical care medicine 36: 1900–1908. doi: 10.1097/CCM.0b013e3181760e5d 18496360 10.1097/CCM.0b013e3181760e5d

[17] Santos CL, Moraes L, Santos RS, Oliveira MG, Silva JD, Maron-Gutierrez T, et al (2012) Effects of different tidal volumes in pulmonary and extrapulmonary lung injury with or without intraabdominal hypertension. Intensive care medicine 38: 499–508. doi: 10.1007/s00134-011-2451-6 22234736 10.1007/s00134-011-2451-6

[18] Baydur A, Behrakis PK, Zin WA, Jaeger M, Milic-Emili J (1982) A simple method for assessing the validity of the esophageal balloon technique. The American review of respiratory disease 126: 788–791. doi: 10.1164/arrd.1982.126.5.788 7149443 10.1164/arrd.1982.126.5.788

[19] Lima RA, Schanaider A, Santana MC, de Oliveira MG, Capelozzi VL, Rocco PR (2011) Developing a new experimental model of abdominal compartment syndrome. Rev Col Bras Cir 38: 417–421. 22267140 10.1590/s0100-69912011000600009

[20] Samary CS, Santos RS, Santos CL, Felix NS, Bentes M, Barboza T, et al (2015) Biological Impact of Transpulmonary Driving Pressure in Experimental Acute Respiratory Distress Syndrome. Anesthesiology 123: 423–433. doi: 10.1097/ALN.0000000000000716 26039328 10.1097/ALN.0000000000000716

[21] Spieth PM, Silva PL, Garcia CS, Ornellas DS, Samary CS, Moraes L, et al (2015) Modulation of stress versus time product during mechanical ventilation influences inflammation as well as alveolar epithelial and endothelial response in rats. Anesthesiology 122: 106–116. doi: 10.1097/ALN.0000000000000415 25141026 10.1097/ALN.0000000000000415

[22] Padilha GA, Horta LF, Moraes L, Braga CL, Oliveira MV, Santos CL, et al (2016) Comparison between effects of pressure support and pressure-controlled ventilation on lung and diaphragmatic damage in experimental emphysema. Intensive Care Med Exp 4: 35 doi: 10.1186/s40635-016-0107-0 27761886 10.1186/s40635-016-0107-0 PMC5071308

[23] Thille AW, Rodriguez P, Cabello B, Lellouche F, Brochard L (2006) Patient-ventilator asynchrony during assisted mechanical ventilation. Intensive Care Med 32: 1515–1522. doi: 10.1007/s00134-006-0301-8 16896854 10.1007/s00134-006-0301-8

[24] Kiss T, Silva PL, Huhle R, Moraes L, Santos RS, Felix NS, et al (2016) Comparison of different degrees of variability in tidal volume to prevent deterioration of respiratory system elastance in experimental acute lung inflammation. Br J Anaesth 116: 708–715. doi: 10.1093/bja/aew093 27106975 10.1093/bja/aew093

[25] Akamine R, Yamamoto T, Watanabe M, Yamazaki N, Kataoka M, Ishikawa M, et al (2007) Usefulness of the 5' region of the cDNA encoding acidic ribosomal phosphoprotein P0 conserved among rats, mice, and humans as a standard probe for gene expression analysis in different tissues and animal species. J Biochem Biophys Methods 70: 481–486. doi: 10.1016/j.jbbm.2006.11.008 17196660 10.1016/j.jbbm.2006.11.008

[26] Schmittgen TD, Lee EJ, Jiang J (2008) High-throughput real-time PCR. Methods Mol Biol 429: 89–98. doi: 10.1007/978-1-60327-040-3_7 18695961 10.1007/978-1-60327-040-3_7

[27] Moraes L, Santos CL, Santos RS, Cruz FF, Saddy F, Morales MM, et al (2014) Effects of sigh during pressure control and pressure support ventilation in pulmonary and extrapulmonary mild acute lung injury. Crit Care 18: 474 doi: 10.1186/s13054-014-0474-4 25113136 10.1186/s13054-014-0474-4 PMC4155110

[28] Matute-Bello G, Downey G, Moore BB, Groshong SD, Matthay MA, Slutsky AS, et al (2011) An official American Thoracic Society workshop report: features and measurements of experimental acute lung injury in animals. Am J Respir Cell Mol Biol 44: 725–738. doi: 10.1165/rcmb.2009-0210ST 21531958 10.1165/rcmb.2009-0210ST PMC7328339

[29] Santos CL, Moraes L, Santos RS, dos Santos Samary C, Silva JD, Morales MM, et al (2014) The biological effects of higher and lower positive end-expiratory pressure in pulmonary and extrapulmonary acute lung injury with intra-abdominal hypertension. Critical care 18: R121 doi: 10.1186/cc13920 24928415 10.1186/cc13920 PMC4095606

[30] Kirkpatrick AW, Roberts DJ, De Waele J, Jaeschke R, Malbrain ML, De Keulenaer B, et al (2013) Intra-abdominal hypertension and the abdominal compartment syndrome: updated consensus definitions and clinical practice guidelines from the World Society of the Abdominal Compartment Syndrome. Intensive care medicine 39: 1190–1206. doi: 10.1007/s00134-013-2906-z 23673399 10.1007/s00134-013-2906-z PMC3680657

[31] Cortes-Puentes GA, Cortes-Puentes LA, Adams AB, Anderson CP, Marini JJ, Dries DJ (2013) Experimental intra-abdominal hypertension influences airway pressure limits for lung protective mechanical ventilation. J Trauma Acute Care Surg 74: 1468–1473. doi: 10.1097/TA.0b013e31829243a7 23694861 10.1097/TA.0b013e31829243a7

[32] Cortes-Puentes GA, Keenan JC, Adams AB, Parker ED, Dries DJ, Marini JJ (2015) Impact of Chest Wall Modifications and Lung Injury on the Correspondence Between Airway and Transpulmonary Driving Pressures. Crit Care Med 43: e287–295. doi: 10.1097/CCM.0000000000001036 26186478 10.1097/CCM.0000000000001036

[33] Kopernik G, Avinoach E, Grossman Y, Levy R, Yulzari R, Rogachev B, et al (1998) The effect of a high partial pressure of carbon dioxide environment on metabolism and immune functions of human peritoneal cells-relevance to carbon dioxide pneumoperitoneum. Am J Obstet Gynecol 179: 1503–1510. 9855588 10.1016/s0002-9378(98)70016-x

[34] Mutoh T, Lamm WJ, Embree LJ, Hildebrandt J, Albert RK (1992) Volume infusion produces abdominal distension, lung compression, and chest wall stiffening in pigs. J Appl Physiol (1985) 72: 575–582. 1559935 10.1152/jappl.1992.72.2.575

[35] Rocco PR, Dos Santos C, Pelosi P (2012) Pathophysiology of ventilator-associated lung injury. Curr Opin Anaesthesiol 25: 123–130. doi: 10.1097/ACO.0b013e32834f8c7f 22395439 10.1097/ACO.0b013e32834f8c7f

[36] Silva PL, Pelosi P, Rocco PR (2016) Optimal mechanical ventilation strategies to minimize ventilator-induced lung injury in non-injured and injured lungs. Expert Rev Respir Med 10: 1243–1245. doi: 10.1080/17476348.2016.1251842 27766893 10.1080/17476348.2016.1251842

[37] Rocco PR, Negri EM, Kurtz PM, Vasconcellos FP, Silva GH, Capelozzi VL, et al (2001) Lung tissue mechanics and extracellular matrix remodeling in acute lung injury. Am J Respir Crit Care Med 164: 1067–1071. doi: 10.1164/ajrccm.164.6.2007062 11587998 10.1164/ajrccm.164.6.2007062

[38] Dolinay T, Kaminski N, Felgendreher M, Kim HP, Reynolds P, Watkins SC, et al (2006) Gene expression profiling of target genes in ventilator-induced lung injury. Physiol Genomics 26: 68–75. doi: 10.1152/physiolgenomics.00110.2005 16569776 10.1152/physiolgenomics.00110.2005

[39] Malbrain ML, Chiumello D, Pelosi P, Wilmer A, Brienza N, Malcangi V, et al (2004) Prevalence of intra-abdominal hypertension in critically ill patients: a multicentre epidemiological study. Intensive Care Med 30: 822–829. doi: 10.1007/s00134-004-2169-9 14758472 10.1007/s00134-004-2169-9

[40] Holodinsky JK, Roberts DJ, Ball CG, Blaser AR, Starkopf J, Zygun DA, et al (2013) Risk factors for intra-abdominal hypertension and abdominal compartment syndrome among adult intensive care unit patients: a systematic review and meta-analysis. Crit Care 17: R249 doi: 10.1186/cc13075 24144138 10.1186/cc13075 PMC4057241

[41] De Waele JJ, Malbrain ML, Kirkpatrick AW (2015) The abdominal compartment syndrome: evolving concepts and future directions. Crit Care 19: 211 doi: 10.1186/s13054-015-0879-8 25943575 10.1186/s13054-015-0879-8 PMC4422424

[42] Pelosi P, Quintel M, Malbrain ML (2007) Effect of intra-abdominal pressure on respiratory mechanics. Acta Clin Belg 62 Suppl 1: 78–88. 10.1179/acb.2007.62.s1.011 24881704

[43] Deeren DH, Dits H, Malbrain ML (2005) Correlation between intra-abdominal and intracranial pressure in nontraumatic brain injury. Intensive Care Med 31: 1577–1581. doi: 10.1007/s00134-005-2802-2 16193329 10.1007/s00134-005-2802-2

[44] Pelosi P, Vargas M (2012) Mechanical ventilation and intra-abdominal hypertension: 'Beyond Good and Evil'. Crit Care 16: 187 doi: 10.1186/cc11874 23256904 10.1186/cc11874 PMC3672607

[45] Regli A, Mahendran R, Fysh ET, Roberts B, Noffsinger B, De Keulenaer BL, et al (2012) Matching positive end-expiratory pressure to intra-abdominal pressure improves oxygenation in a porcine sick lung model of intra-abdominal hypertension. Crit Care 16: R208 doi: 10.1186/cc11840 23098278 10.1186/cc11840 PMC3682312

[46] Regli A, Chakera J, De Keulenaer BL, Roberts B, Noffsinger B, Singh B, et al (2012) Matching positive end-expiratory pressure to intra-abdominal pressure prevents end-expiratory lung volume decline in a pig model of intra-abdominal hypertension. Crit Care Med 40: 1879–1886. doi: 10.1097/CCM.0b013e31824e0e80 22488004 10.1097/CCM.0b013e31824e0e80

[47] Spieth PM, Carvalho AR, Guldner A, Kasper M, Schubert R, Carvalho NC, et al (2011) Pressure support improves oxygenation and lung protection compared to pressure-controlled ventilation and is further improved by random variation of pressure support. Crit Care Med 39: 746–755. doi: 10.1097/CCM.0b013e318206bda6 21263322 10.1097/CCM.0b013e318206bda6

[48] Guldner A, Braune A, Carvalho N, Beda A, Zeidler S, Wiedemann B, et al (2014) Higher levels of spontaneous breathing induce lung recruitment and reduce global stress/strain in experimental lung injury. Anesthesiology 120: 673–682. doi: 10.1097/ALN.0000000000000124 24406799 10.1097/ALN.0000000000000124

[49] Carvalho NC, Guldner A, Beda A, Rentzsch I, Uhlig C, Dittrich S, et al (2014) Higher levels of spontaneous breathing reduce lung injury in experimental moderate acute respiratory distress syndrome. Crit Care Med 42: e702–715. doi: 10.1097/CCM.0000000000000605 25162475 10.1097/CCM.0000000000000605

[50] Pellegrini M, Hedenstierna G, Roneus A, Segelsjo M, Larsson A, Perchiazzi G (2016) The Diaphragm Acts as a Brake During Expiration to Prevent Lung Collapse. Am J Respir Crit Care Med. 10.1164/rccm.201605-0992OC 27922742

[51] Wrigge H, Zinserling J, Neumann P, Defosse J, Magnusson A, Putensen C, et al (2003) Spontaneous breathing improves lung aeration in oleic acid-induced lung injury. Anesthesiology 99: 376–384. 12883410 10.1097/00000542-200308000-00019

[52] Wrigge H, Zinserling J, Neumann P, Muders T, Magnusson A, Putensen C, et al (2005) Spontaneous breathing with airway pressure release ventilation favors ventilation in dependent lung regions and counters cyclic alveolar collapse in oleic-acid-induced lung injury: a randomized controlled computed tomography trial. Crit Care 9: R780–789. doi: 10.1186/cc3908 16356227 10.1186/cc3908 PMC1414014

[53] Carvalho AR, Spieth PM, Pelosi P, Beda A, Lopes AJ, Neykova B, et al (2009) Pressure support ventilation and biphasic positive airway pressure improve oxygenation by redistribution of pulmonary blood flow. Anesth Analg 109: 856–865. doi: 10.1213/ane.0b013e3181aff245 19690258 10.1213/ane.0b013e3181aff245

[54] Yoshida T, Uchiyama A, Matsuura N, Mashimo T, Fujino Y (2012) Spontaneous breathing during lung-protective ventilation in an experimental acute lung injury model: high transpulmonary pressure associated with strong spontaneous breathing effort may worsen lung injury. Critical care medicine 40: 1578–1585. doi: 10.1097/CCM.0b013e3182451c40 22430241 10.1097/CCM.0b013e3182451c40

[55] Yoshida T, Fujino Y, Amato MB, Kavanagh BP (2017) Fifty Years of Research in ARDS. Spontaneous Breathing during Mechanical Ventilation. Risks, Mechanisms, and Management. American journal of respiratory and critical care medicine 195: 985–992. doi: 10.1164/rccm.201604-0748CP 27786562 10.1164/rccm.201604-0748CP

[56] Yoshida T, Torsani V, Gomes S, De Santis RR, Beraldo MA, Costa EL, et al (2013) Spontaneous effort causes occult pendelluft during mechanical ventilation. American journal of respiratory and critical care medicine 188: 1420–1427. doi: 10.1164/rccm.201303-0539OC 24199628 10.1164/rccm.201303-0539OC

[57] Roberts DJ, Ball CG, Kirkpatrick AW (2016) Increased pressure within the abdominal compartment: intra-abdominal hypertension and the abdominal compartment syndrome. Curr Opin Crit Care 22: 174–185. doi: 10.1097/MCC.0000000000000289 26844989 10.1097/MCC.0000000000000289

[58] Grasso F, Engelberts D, Helm E, Frndova H, Jarvis S, Talakoub O, et al (2008) Negative-pressure ventilation: better oxygenation and less lung injury. Am J Respir Crit Care Med 177: 412–418. doi: 10.1164/rccm.200707-1004OC 18079496 10.1164/rccm.200707-1004OC

[59] Guldner A, Braune A, Ball L, Silva PL, Samary C, Insorsi A, et al (2016) Comparative Effects of Volutrauma and Atelectrauma on Lung Inflammation in Experimental Acute Respiratory Distress Syndrome. Crit Care Med 44: e854–865. doi: 10.1097/CCM.0000000000001721 27035236 10.1097/CCM.0000000000001721 PMC5105831

[60] Neumann P, Wrigge H, Zinserling J, Hinz J, Maripuu E, Andersson LG, et al (2005) Spontaneous breathing affects the spatial ventilation and perfusion distribution during mechanical ventilatory support. Critical care medicine 33: 1090–1095. 15891341 10.1097/01.ccm.0000163226.34868.0a

[61] Blanch L, Villagra A, Sales B, Montanya J, Lucangelo U, Lujan M, et al (2015) Asynchronies during mechanical ventilation are associated with mortality. Intensive Care Med 41: 633–641. doi: 10.1007/s00134-015-3692-6 25693449 10.1007/s00134-015-3692-6

[62] Henzler D, Hochhausen N, Bensberg R, Schachtrupp A, Biechele S, Rossaint R, et al (2010) Effects of preserved spontaneous breathing activity during mechanical ventilation in experimental intra-abdominal hypertension. Intensive Care Med 36: 1427–1435. doi: 10.1007/s00134-010-1827-3 20237763 10.1007/s00134-010-1827-3

[63] Regli A, Mahendran R, Fysh ET, Roberts B, Noffsinger B, De Keulenaer BL, et al (2012) Matching positive end-expiratory pressure to intra-abdominal pressure improves oxygenation in a porcine sick lung model of intra-abdominal hypertension. Critical care 16: R208 doi: 10.1186/cc11840 23098278 10.1186/cc11840 PMC3682312

[64] Ke L, Tong ZH, Ni HB, Ding WW, Sun JK, Li WQ, et al (2012) The effect of intra-abdominal hypertension incorporating severe acute pancreatitis in a porcine model. PloS one 7: e33125 doi: 10.1371/journal.pone.0033125 22403734 10.1371/journal.pone.0033125 PMC3293917

