# Supplementary material for: Effects of pressure support and pressure-controlled ventilation on lung damage in a model of mild extrapulmonary acute lung injury with intra-abdominal hypertension
Source: PLoS One. 2017 May 25;12(5):e0178207. doi: 10.1371/journal.pone.0178207 (PMC5444773; doi:10.1371/journal.pone.0178207)
Supplement: S1 Table — Primers used in experiments. IL-6, interleukin-6; PCIII, pro-collagen III; SP-B, surfactant protein B; VCAM-1, vascular cell adhesion molecule-1; 36B4, acidic ribosomal phosphoprotein P0. (DOCX) [file pone.0178207.s001.docx]

**S1 Table**. Forward and reverse oligonucleotide sequences of target gene primers

| **Gene** | **Primer** | **Primer sequences (5′-3′)** |
| --- | --- | --- |
| IL-6 | Forward | CTC CGC AAG AGA CTT CCA G |
|  | Reverse | CTC CTC TCC GGA CTT GTG A |
| PCIII | Forward | ACC TGG ACC ACA AGG ACA |
|  | Reverse | TGG ACC CAT TTC ACC TTT |
| Amphiregulin | Forward | TTT CGC TGG CGC TCT CA |
|  | Reverse | TTC CAA CCC AGC TGC ATA ATG |
| SP-B | Forward | CCA TCC CTC TGC CCT TCT G |
|  | Reverse | CAC CCT TGG GAA TCA CAG CTT |
| VCAM-1 | Forward | TGC ACG GTC CCT AAT GTG TA |
|  | Reverse | TGC CAA TTT CCT CCC TTA AA |
| 36B4 | Forward | AAT CCT GAG CGA TGT GCA G |
|  | Reverse | GCT GCC ATT GTC AAA CAC |

Primers used in experiments. IL-6: interleukin-6; PCIII: type III procollagen; Amphiregulin; SP-B: surfactant protein B; VCAM-1: vascular cell adhesion molecule one.
