# Supplementary material for: Effects of pressure support and pressure-controlled ventilation on lung damage in a model of mild extrapulmonary acute lung injury with intra-abdominal hypertension
Source: PLoS One. 2017 May 25;12(5):e0178207. doi: 10.1371/journal.pone.0178207 (PMC5444773; doi:10.1371/journal.pone.0178207)
Supplement: S2 Table — (DOCX) [file pone.0178207.s002.docx]

**S2 Table.** Amount of fluids administered and mean arterial pressure

|  |  | nIAP | | IAH | |
| --- | --- | --- | --- | --- | --- |
|  | Time point | PCV | PSV | PCV | PSV |
| Ringer’s Lactate (mL) | Start | 0.0 ± 0.0 | 0.0 ± 0.0 | 0.0 ± 0.0 | 0.0 ± 0.0 |
|  | End | 4.5 ± 0.9 | 5.0 ± 1.5 | 6.3 ± 0.9 | 4.3 ± 0.3 |
| Colloid (mL) | Start | 0.0 ± 0.0 | 0.0 ± 0.0 | 0.0 ± 0.0 | 0.0 ± 0.0 |
|  | End | 3.9 ± 2.3 | 0.8 ± 0.9# | 5.9 ± 2.5 | 2.0 ± 1.8# |
| MAP (mmHg) | Start | 73 ± 9 | 141 ± 28# | 83 ± 24 | 114 ± 32# |
|  | End | 71 ± 21 | 88 ± 20 | 78 ± 26 | 102 ± 24 |

IAH, intra-abdominal hypertension; nIAP, normal intra-abdominal pressure; PCV, pressure-controlled ventilation; PSV, pressure support ventilation; Start, after sham surgery (nIAP) or IAH induction; End, nIAP or IAH after 1 h mechanical ventilation with PCV or PSV; MAP, mean arterial pressure. Comparisons were performed by two-way repeated-measures ANOVA followed by Bonferroni’s post-hoc test (p < 0.05). Values are given as mean ± standard deviation of 6 animals/group. #Significantly different from PCV group at the corresponding time point (p<0.05).
