# Supplementary material for: Effects of pressure support and pressure-controlled ventilation on lung damage in a model of mild extrapulmonary acute lung injury with intra-abdominal hypertension
Source: PLoS One. 2017 May 25;12(5):e0178207. doi: 10.1371/journal.pone.0178207 (PMC5444773; doi:10.1371/journal.pone.0178207)
Supplement: S1 Fig — Original magnification ×200. Arrows: alveolar collapse. AD: alveolar duct. nIAP: animals with normal intra-abdominal pressure. IAH: animals with intra-abdominal hypertension. PCV: mechanically ventilated in pressure-controlled ventilation. PSV: mechanically ventilated with pressure support ventilation. NV: non-ventilated animals. (DOCX) [file pone.0178207.s003.docx]

**S1 Figure: Photomicrographs of lung parenchyma stained with hematoxylin-eosin.**


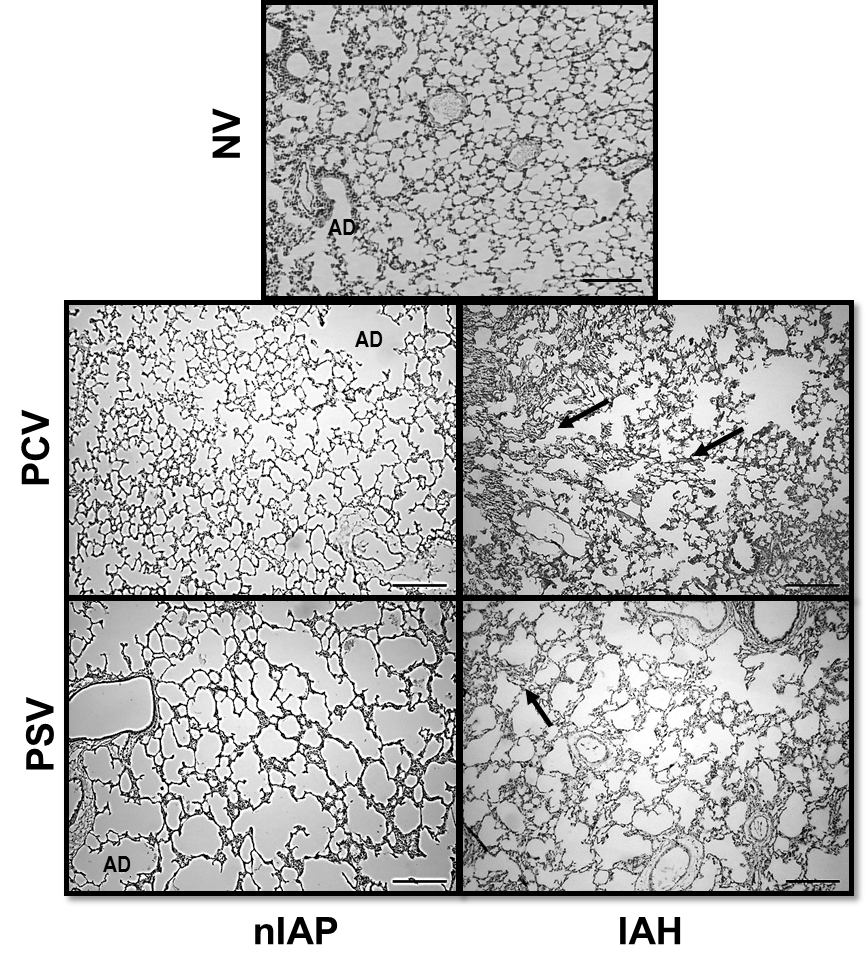


Original magnification ×200. Arrows: alveolar collapse. AD: alveolar duct. nIAP: animals with normal intra-abdominal pressure. IAH: animals with intra-abdominal hypertension. PCV: mechanically ventilated in pressure-controlled ventilation. PSV: mechanically ventilated with pressure support ventilation. NV: non-ventilated animals.
